# A Case of Excision of the Cervix Uteri

**Published:** 1867-09

**Authors:** 


### A CASE OF EXCISION OF THE CERVIX UTERI.

In recently looking over an old volume of Surgery, (*The Art of Surgery, etc.*, by Daniel Turner, M.D., London, 1736,) we came across the following interesting case. The operation performed on the patient herself, is evidently the same as that recently proposed by Huguier for so-called procidentia, and which is now attracting so much attention. The case is narrated under the heading "Of a Procidentia Uteri, ac Prolapsus Vaginæ ejus." We transcribe it entire, believing it will be of interest to the profession, not alone on account of its novelty and the quaintness of the narrative, but also from the fact that it is probably the first case on record of this operation for the cure of procidentia:

An elderly woman, having been for some years crazy, as well in her head, I mean her intellect, as her body, and longer incommoded with a procidentia uteri, which she was forced to keep up with her string-cloth, being otherwise scarce able to move about the house; under a fit of melancholy, was pondering how to free herself from this inconvenience, and unknown to any person of the family, taking her opportunity, first putting herself in a suitable posture, with one hand she draws down the prolapsed body, whilst with her husband's razor in the other, got as it were, by stealth, she excised all within her reach; then, putting a clout up to the parts, she got into her bed; where, after short time the blood being discovered, and she questioned about it, she very sedately told them what she had done.

Upon this a neighboring surgeon was sent for, who restrained the hæmorrhage with proper restringents: but in the evening, the flux being renewed, he called me to his assistance: when, ordering a large tent, like a pessus, to be made up, the same was dipped in oxycrat. cum alb. ovorum, then gently expressed and rolled over some fine bole, particularly its extremity, and so put up; next to this, a thick pledget, or wad of tow, wrung out of the same, and sprinkled thick cum farina fabar, with the T-bandage to keep all close.

The day following, the blood seemingly staunched, a digestive was got ready, into which made warm, a tent like the first was dipped and introduced: but coming the third day, we found more blood discharged, several grumes, or clots, following the extraction of this last application; so that we were forced to have recourse to our restringents: and thus we continued for several days, our patient all this while calm, with little fever, and as little complaint, in regard to what might have been expected from so desperate an operation.

When the flux was stopped, and the external privity cleansed with a warm stuph wrung out of wine, we took a more strict survey of the parts, and dilating the labia with my fingers, in expectation of finding a wound on the relaxed vagina, could perceive nothing like it, all lying fair and natural within our sight: when, entering my finger as high up as I could reach, I plainly felt a large jagged or unequal wound, on the lower part of the uterus, whose os internum, or whole cervix, had been cut off: the blood, upon this examination (though but in small quantity) again following my finger.

I then inquired after the part thus strangely taken off, which they shewed me put by in water, and I perceived it, as I have represented, the upper part an inch and a half deep, especially on each side, somewhat narrower in the middle, and still less on the under part, or that lying on the rectum, which, for the curiosity, I desired the surgeon to put into spirit of wine, and which he still keeps by him, to gratify the ingenuous inquirer with a sight thereof; of which number the first person I showed it to was Mr. Petty, in Fenchurch street: but, proceeding,

After this discovery, the blood also entirely restrained, I advised a large tent to be made up, as at first, whose upper extremity was dipped in a melted mixture, ex part. iij., vel cerciter, linimenti arcæi, cum quarta ol. tereb., passing it up against the gaping wound of the uterus; I also advised a warm fomentation, which we were shy of sooner, on account of the hæmorrhage, prepared ex Decocto fol. absinth. cent. hyper, etc., and a proportionate quantity of the spir. vin. camph. to be applied with stuphs, not only to the pudendum, but reaching up to the lower belly, which were renewed for half an hour, night and morning, before the time of dressing up, whereby to comfort the internal parts thereof, cherish their heat, and promote digestion of the wound; which, after ten days, began to appear laudable upon the end of the tent, and in moderate quantity.

After this a womb-syringe was provided, and the following decoction thrown in twice a day, by way of injection, to mundify the wound; by its situation I apprehended it less susceptible of an impression of our balsams, which were, however, still continued after the use thereof.

℞.—Plantag. cum toto, summit. hyperic. centaur, ana, m. j. hord. gallic. ℥ ss. coquantur in aq. font. q. s. pro ℔i. colaturæ, cui per subsidentiam depuratæ adde mel rosar. ℥ij. tinc. myrrh. ℥ ss. et f. mixtura, cujus, metrenchitæ auxilio, injiciantur cochlear. v. vel vi. per sinum Pudoris, prius tepefacta, bis in die.

This having been used for some days, and the discharge still lessening, I substituted the following, more consolidating and agglutinating:

℞.—Rad. de symphyto, plantag. ana ℥i. fol. hyperic. equiseti, saniculæ, bugulæ, ana m. ss. coquantur in aq. font. q. s. ad ℥xij. colaturæ, sub sinem infundendo vini rub. ℥iv. et preterea colature suprascriptœ addendo mel rosar. ℥ij. f. pro injectione prioris instar utenda, sed sæpius in die.

By which I have great hopes her cure may be accomplished; she now gets out of bed, takes her nourishment and rest, the discharge from the wound being inconsiderable; and the same bidding fair for healing suddenly.

I think this is, if not the only instance of the *niseratomia*, yet surely of the uteri cervicis abscissio, I remember to have met with in our writers of chirurgery; at least I am apt to believe the first attempt at this way, for the cure of its prolapsus. —*Journal of Practical Medicine and Surgery.*

